# Optimizing care in osteoporosis: The Canadian quality circle project

**DOI:** 10.1186/1471-2474-9-130

**Published:** 2008-10-01

**Authors:** George Ioannidis, Lehana Thabane, Amiram Gafni, Anthony Hodsman, Brent Kvern, Dan Johnstone, Nathalie Plumley, Lena Salach, Famida Jiwa, Jonathan D Adachi, Alexandra Papaioannou

**Affiliations:** 1 Department of medicine, McMaster University, Hamilton, Ontario, Canada; 2 Department of clinical epidemiology and biostatistics, McMaster University, Hamilton, Ontario, Canada; 3 Director of biostatistics at the Father Sean O'Sullivan Research Centre and Centre for Evaluation of Medicines at St Joseph's Healthcare in Hamilton, Hamilton, Ontario Canada; 4 Department of medicine, University of Western Ontario, London, Ontario, Canada; 5 Department of family medicine, University of Manitoba, Winnipeg, Manitoba, Canada; 6 Procter & Gamble Pharmaceuticals, Toronto, Ontario, Canada; 7 Research and Professional Development, Ontario College of Family Physicians, Toronto, Ontario, Canada; 8 Osteoporosis Canada, Toronto, Ontario, Canada

## Abstract

**Background:**

While the Osteoporosis Canada 2002 Canadian guidelines provided evidence based strategies in preventing, diagnosing, and managing this condition, publication and distribution of guidelines have not, in and of themselves, been shown to alter physicians clinical approaches. We hypothesize that primary care physicians enrolled in the Quality Circle project would change their patient management of osteoporosis in terms of awareness of osteoporosis risk factors and bone mineral density testing in accordance with the guidelines.

**Methods:**

The project consisted of five Quality Circle phases that included: 1) Training & Baseline Data Collection, 2) First Educational Intervention & First Follow-Up Data Collection 3) First Strategy Implementation Session, 4) Final Educational Intervention & Final Follow-up Data Collection, and 5) Final Strategy Implementation Session. A total of 340 circle members formed 34 quality circles and participated in the study. The generalized estimating equations approach was used to model physician awareness of risk factors for osteoporosis and appropriate utilization of bone mineral density testing pre and post educational intervention (first year of the study). Odds ratios (OR) and 95% confidence intervals (95% CI) were calculated.

**Results:**

After the 1^st ^year of the study, physicians' certainty of their patients' risk factor status increased. Certainty varied from an OR of 1.4 (95% CI: 1.1, 1.8) for prior vertebral fracture status to 6.3 (95% CI: 2.3, 17.9) for prior hip fracture status. Furthermore, bone mineral density testing increased in high risk as compared with low risk patients (OR: 1.4; 95% CI: 1.2, 1.7).

**Conclusion:**

Quality Circle methodology was successful in increasing both physicians' awareness of osteoporosis risk factors and appropriate bone mineral density testing in accordance with the 2002 Canadian guidelines.

## Background

Approximately one in four women have osteoporosis and a 50-year old white woman has a remaining lifetime risk of 40% for sustaining a hip, vertebra or a wrist fracture [1,2]. These fractures have physical, psychological, social and economic consequences that can profoundly influence health related quality of life [3-7]. With the expectation that the aging population will increase in subsequent years [8], it is predicted that increased rates of osteoporosis will also occur making it a major public health concern worldwide. Given the millions of women who have or will develop osteoporosis, the detection of the disease must become as familiar to family physicians as the detection of hypertension and diabetes.

Despite the high prevalence of this disease, there is evidence that patients at high risk of fracture due to osteoporosis are not being diagnosed or treated with appropriate therapies [9-12]. While the Osteoporosis Canada 2002 Canadian guidelines [9] provided evidence based strategies in managing this condition, publication and distribution of guidelines have not, in and of themselves, been shown to alter physicians' clinical approaches [13]. Therefore, a gap exists between care delivery and best known practices in the management of osteoporosis. The Canadian Quality Circle (CQC) Project, a multifaceted integrated disease management process strategy utilizing reflective learning approaches [14,15] was developed and implemented to reduce this care gap. We hypothesize that primary care physicians' perceived awareness of osteoporosis risk factors in their patients and bone mineral density testing would change in accordance with the Osteoporosis Canada guidelines as a result of their enrollment in the study.

## Methods

### Physician recruitment

All participating physicians provided written informed consent. The study was approved by Health Research Ethics Boards across Canada. The study was sponsored by research grants from the Ontario College of Family Physicians and Alliance for Better Bone Health (Procter & Gamble Pharmaceuticals Canada Inc. and Sanofi-Aventis Pharma Inc.).

#### Facilitators

A physician-facilitator was selected for each Quality Circle and trained to facilitate the management of the circle. Facilitators were local family physicians recruited to lead and initiate discussion at study meetings and were chosen because of their skills in small group facilitation and involvement in continuing professional development and were selected by the study's steering committee.

#### Osteoporosis specialist

An osteoporosis specialist was assigned to each quality circle. The specialist was recruited on the recommendation provided by the facilitator from the facilitator's local referral network. The role of the osteoporosis specialist was to attend each Quality Circle meeting to provide assistance in addressing clinical matters.

#### Members

Circle members were family physicians selected from specific geographical regions across Canada derived from a list developed by the facilitator of each Quality Circle, supplemented with physicians' names from the provincial College of Family Physician's membership list. All potential members received a written introductory letter from the local circle facilitator and were invited to participate in the study. A maximum of 15 physicians in each geographical area were enrolled into each circle in the study. Family physicians are defined as physicians who take professional responsibility for the comprehensive care of unselected patients with undifferentiated problems and are committed to the person regardless of gender, age, or illness [16].

### Overview of project phases

**Figure 1:**
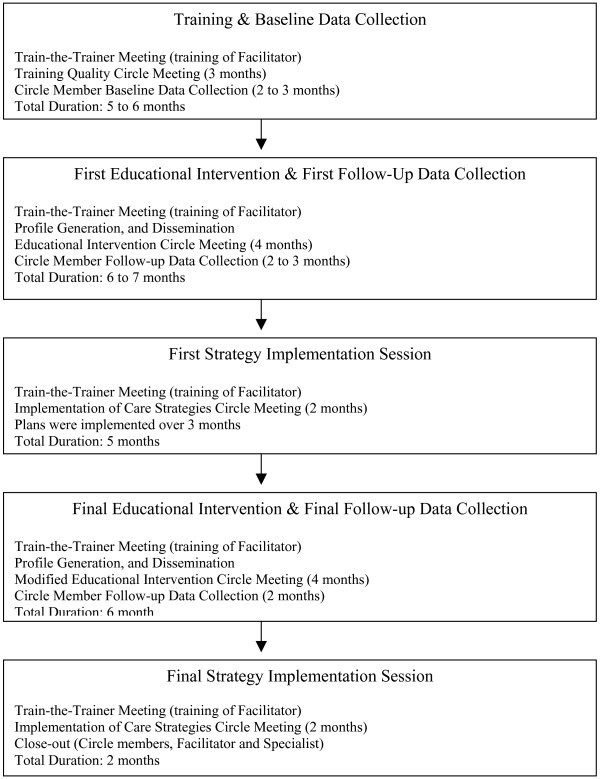
**Five phases of the Canadian quality circle project.** 1) Introduction, Training & Baseline Data Collection, 2) First Educational Intervention & First Follow-Up Data Collection 3) First Strategy Implementation Session, 4) Final Educational Intervention & Final Follow-up Data Collection, and 5) Final Strategy Implementation Session & final follow-up data collection (Figure 1). Members committed to the first year of the project, which consist of Quality Circle phases one to three. Those who were interested and willing to commit to the second year of the project completed the remaining project phases (four and five).

The overall project consisted of five Quality Circle phases. Members committed to the first year of the project, which consist of Quality Circle phases one to three. Those who were interested and willing to commit to the second year of the project completed the remaining 2 project phases. The five phases include: 1) Introduction, Training & Baseline Data Collection, 2) First Educational Intervention & First Follow-Up Data Collection 3) First Strategy Implementation Session, 4) Final Educational Intervention & Final Follow-up Data Collection, and 5) Final Strategy Implementation Session (Figure 1). Participants received continuing professional development credits from the College of Family Physicians of Canada (12 MAINPRO-C credits (or 24 MAINPRO-M1 credits)) per year for participation.

This article describes the changes in physician behaviour during the first year of the project, but the over-all timeline of the Project is summarized in Figure 1, and the detailed description of all 5 phases follows.

#### Phase 1: introduction, training & baseline data collection

Project training was conducted via baseline circle meetings and involved a systematic examination of the study protocol, and a review of the project data collection form. Following training, each member collected baseline patient data from their own practice using a standardized baseline data collection form to ascertain current practice patterns for the diagnosis and treatment of osteoporosis. Once the data were evaluated, the Steering Committee met to review findings and determine key learnings that circle members should know. Key learnings were used to produce Physician Profiles. Profiles are "snapshots" of members' practice patterns showing how they managed osteoporosis including risk factor identification, bone mineral density testing, and therapies. Physician profiles were displayed graphically with a brief text summary. The profiles permitted anonymous comparisons of individual circle member data with their peers in their circle and with all the participating physicians in the project.

#### Phase 2: first educational intervention & first follow-up data collection

The educational intervention was initiated through a phase 2 Quality Circle meeting. The intervention consisted of two parts, 1) the presentation and discussion of baseline individual, group and overall Quality Circle Profiles. The profiles were provided to the members on-site. Then 2) educational materials related to the 2002 Osteoporosis Canada guidelines were distributed, discussed and an educational workshop was conducted. The facilitators led group discussions with their circles to identify barriers in managing osteoporosis as suggested by the guidelines and strategies to improve patient care. Following the educational intervention, a second phase of data collection was conducted by each member on additional patients using the follow-up Quality Circle collection form. Following the data collection period, the Steering Committee reviewed the findings, generated key learnings, and determined the next intervention.

#### Phase 3: first strategy implementation session

A strategy implementation meeting was conducted comparing individual, group and overall Quality Circle profiles from the baseline and first follow-up data collection periods. Discussions concerning the progress made by incorporating strategies identified in the prior phases of the project were shared among the group. Based on the major findings from the profiles, members discussed additional measures that should be implemented in their practices to increase alignment with the 2002 guidelines.

#### Phase 4: final educational intervention & final follow-up data collection

The final Quality Circle educational intervention meetings – phase 4 – were similarly designed to the first educational intervention meetings. However, the educational intervention focused on areas where the data showed physicians demonstrated suboptimal knowledge regarding the appropriate management of osteoporosis according the 2002 guidelines. Following the second educational intervention, data were collected by each physician on additional patients using the Quality Circle collection form.

#### Phase 5: final strategy implementation session

The final phase 5 strategy implementation meetings were similarly designed to the first strategy implementation meetings.

### Procedures for data collection

#### Criteria for patient selection, screening & completion of the quality circle collection forms

Eligible patients met the following criteria: women 55 years of age and older, known to the physician, and attended at least two visits to the physician's clinic in the 24 months prior to enrollment. The screening of eligible patients was conducted by the clinic nurse to overcome the possibility of physician bias. At the end of each recruitment day, the nurse used the day's visit schedule to randomly select three or four medical charts of patients that met the study's eligibility criteria. After making the selection, the nurse placed the patient questionnaire into each patient chart and the family physician completed the form. Each form was one page and took approximately 5 minutes to complete. All forms were faxed to a central site and the information was incorporated in an electronic database for analysis. For each data collection period (phases one, two and four), a total of 25 different patients were randomly selected for evaluation. Over the course of the study, the forms were slightly modified to better capture important clinical data.

### Multifaceted educational intervention

The educational intervention consisted of eight key components: 1) audit and feedback, where standardized Quality Circle Data Collection Forms were used to audit physicians' practices and physicians profiles were generated to provide feedback; 2) interactive small group discussions at all 5 Quality Circle meetings, where participants could safely reflect on their own practice patterns compared to their peers and compared to a gold standard; 3) use of opinion leaders who were local primary care physicians who not only served as meeting facilitators but as peers who thought the information being discussed was important; 4) reminders, where the standardized collection forms, being filled out repeatedly over a number of weeks, triggered thought on a patient's risk factors for fracture, bone mineral density utilization, and therapies; 5) multi-professional collaboration and community building where osteoporosis specialists attended each Quality Circle meeting to provide assistance in addressing clinical matters but also become personally known by circle members; 6) financial intervention of $10 for each completed standardized patient form; 7) patient directed interventions where the primary care physicians distributed Osteoporosis Canada information and educational tools for patient use; 8) and educational workshops built on needs assessments of the participants as defined by the data collected from participants own practices and focus group feedback from a cross-section of circle members. These interventions have been shown to be effective in changing behaviour [17-21].

A series of five Quality Circle Educational Intervention Workshops were developed by the Core Educational Committee consisting of members of the Osteoporosis Canada, Ontario College of Family physicians, leading physicians and scientists, and industry partners. The 2002 Osteoporosis Canada guidelines were used as the main evidence-based reference for the workshops. The workshops were developed to meet the identified needs of the cohort and required 60–90 minutes to administer during the circle meetings.

### Risk factor assessment and bone densitometry

According to the Osteoporosis Canada 2002 guidelines, all postmenopausal women over the age of 50 years should be assessed for the presence of risk factors for osteoporosis. A patient was considered to be at high risk if they had one major or 2 or more minor risk factors for fracture. Low risk patients had at most one minor risk factor for fracture [9].

For individuals under the age of 65 years, a bone mineral density measurement is recommended for those who have at least one major, or 2 minor risk factors for future fracture (high risk). In addition, all women 65 years of age and older should have a bone mineral density test conducted because of the high risk of osteoporosis and fracture in this group [9].

### Statistical analysis

The generalized estimating equations [22] approach was used to model differences in physician perceived certainty of risk factors for osteoporosis and appropriate utilization of bone mineral density testing pre and post educational intervention (first year of the study). An exchangeable correlation matrix was used for the analyses. Physicians' awareness of the following risk factors were examined: age 65 years and older; prior fragility fracture after age 40 years at the hip wrist, or spine; family history of fracture; menopause before age 45 years; any other major risk factor; and two or more minor risk factors. Generalized estimating equations method was used to take into account the clustered nature of the data; given that patients treated within a primary care physician should be correlated (clustered variable is the physician). For the model, the unit of analysis is the patient and the unit of inference is the physician. Separate models were conducted for the dependent variables, risk factor certainty and appropriate bone mineral density testing. Unadjusted and adjusted odds ratios (OR) and corresponding 95% confidence intervals (CI) are reported.

A physician was certain about a patient's particular risk factor if the physician indicate on the standardized form that the patient had (yes) or did not have (no) the risk factor. If the physician was unsure, the physician indicated uncertain on the standardized form. Appropriate bone mineral density testing was defined, based on the Osteoporosis Canada guidelines, as testing in those who have at least one major, or 2 minor risk factors for future fracture (high risk) or not testing in patients with one minor or no risk factors. Given that only those who were interested and willing to commit to the second year of the project completed year two of the study, the analyses were conducted for only the first year. Goodness-of-fit of each model was assessed using the method developed by Horton et al. [23].

All statistical analyses were performed using the SAS/STAT (version 9.1; SAS Institute Inc., Cary, North Carolina, USA) software package running on Windows XP Professional. The criterion for statistical significance was set at α 0.05.

## Results

A total of 340 family physician circle members formed 34 quality circles and participated in the study. During the first year of the study, 39 physicians (11.5%) dropped out of the study (Table 1). Quality circles were developed in seven provinces including 5 in British Columbia, 3 in Alberta, 1 in Saskatchewan, 11 in Ontario, 11 in Quebec, 1 in New Brunswick, 1 in Nova Scotia. Bone mineral density testing was conducted in 66% (5549/8371) and 74% (5431/7328) of the patient population at baseline and the first follow-up, respectively.

**Table 1:** Study and patient characteristics of the quality circle project

	**Baseline**	**1^st ^Follow-up**
***Study characteristics: n***		
Number of provinces	7	7
Number of cities	109	97
Number of circles	34	34
Number of physicians	340	301
Number of chart reviews	8376	7354
		
*Risk factors for fracture: n with characteristic/total n (%)*		
Age ≥ 65 yrs	5337/8362 (63.8)	4440/7341 (60.5)
Prior hip fracture	152/8366 (1.8)	187/7344 (2.6)
Prior wrist fracture	353/8367 (4.2)	393/7345 (5.3)
Prior vertebral fracture	486/8365 (5.8)	481/7339 (6.6)
Family history of fracture	431/8368 (5.2)	649/7337 (8.9)
At least 1 fall in the previous 12 months	926/8365 (11.1)	NA*
Oral Prednisone therapy (> 3 months)	353/8366 (4.2)	NA*
Menopause before age 45 yrs	689/8364 (8.2)	643/7339 (8.8)
Any other major risk factor	3485/8364 (41.7)	1166/7329 (25.1)
Two or more minor risk factors	1467/8356 (17.6)	1378/7326 (18.8)
High risk	6486/8376 (77.4)	5569/7354 (75.7)
		
Bone density testing: n with characteristic (%)	n = 8371	n = 7328
No test	2822 (33.7)	1897 (25.9)
T-score: >-1	1490 (17.5)	1295 (17.7)
T-score: -1 to -2.5	2028 (24.2)	2282 (31.4)
T-score: <-2.5	1774 (21.2)	1272 (17.4)
Test results pending	257 (3.1)	582 (7.9)

*NA = not available (it was not measured during follow-up).

### Awareness of risk factors

**Figure 2:**
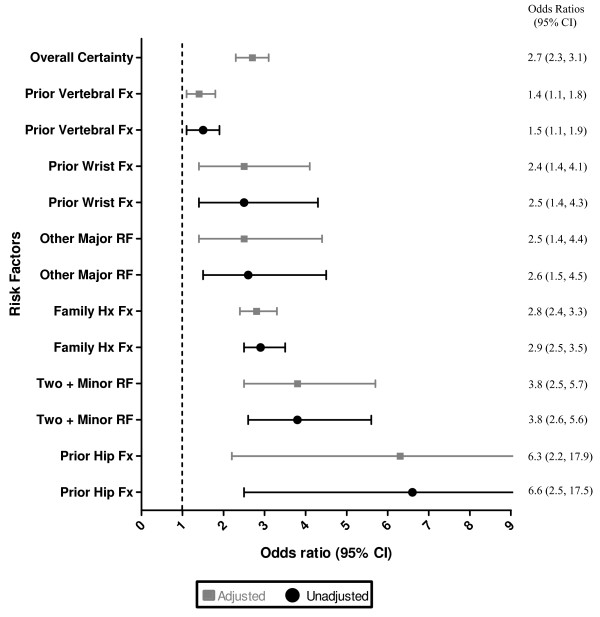
**Change in physicians' perceived certainty of their patients' risk factor status during the 1^st ^year of the study.** The generalized estimating equations approach was used to model differences in physician perceived awareness of risk factors pre and post 1^st ^educational intervention. Values are expressed as adjusted and unadjusted odds ratios (OR) and corresponding 95% confidence intervals (CI) are reported. Generalized estimating equations method was used to take into account the clustered nature of the data (patients within physicians). All risk factors in figure 2 were included in the adjusted analysis. Overall Certainty = all the risk factors in figure 2 combined. UP = upper. The fit of the models ranged from 0.714 to < 0.001.

The percentage of primary care physicians who were uncertain of their patients' risk factor status was generally low. However, at baseline, approximately 50% (4238/8368), 22% (1794/8364) and 10% (823/8365) of physicians professed uncertainty about three key historical facts: their patients' family history of fracture, early menopausal status, and prior vertebral fracture history, respectively (Table 2). Results generated from the generalized estimating equations method showed that physicians' certainty of risk factor awareness significantly increased during the first year of the study. This implies that more physicians indicated that they were aware of the patients' risk factor status (either the patient had or did not have the risk factor) then were unaware during the course of the study. Improvement varied from an OR of 1.4 (CI: 1.1, 1.8) for prior vertebral fracture status to 6.5 (CI: 2.4, 17.5) for prior hip fracture status (Figure 2).

**Table 2:** Physicians' perceived uncertainty of their patients risk factor status

Patient characteristics: n uncertain/total n (%)	Baseline	1^st ^Follow-up n (%)
Age ≥ 65 yrs	0/8362 (0.0)	1/7341(< 0.1)
Prior hip fracture	151/8366 (1.8)	23/7344 (0.3)
Prior wrist fracture	238/8367 (2.8)	93/7345 (1.3)
Prior vertebral fracture	823/8365 (9.8)	537/7339 (7.3)
Family history of fracture	4238/8368 (50.7)	1939/7337 (26.4)
At least 1 fall in the previous 12 months	853/8365 (10.2)	NA*
Oral Prednisone therapy (> 3 months)	92/8366 (1.1)	NA*
Menopause before age 45 yrs	1794/8364 (21.5)	835/7339 (11.4)
Any other major risk factor	176/8364 (2.1)	63/7329 (0.9)
Two or more minor risk factors	518/8356 (6.2)	131/7326 (1.8)

Physician's check off the uncertain option on the standardized form for the above patient characteristics (physicians were unaware whether their patients had this characteristic). *NA = not available (it was not measured during follow-up).

### Appropriate bone mineral density testing

**Figure 3:**
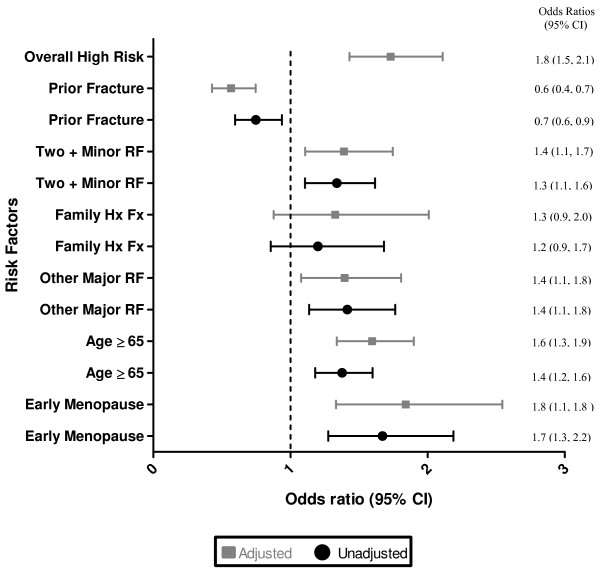
**Change in bone mineral density testing depending of risk factor status during the 1^st ^year of the study.** The generalized estimating equations approach was used to model differences in appropriate utilization of bone mineral density testing pre and post 1^st ^educational intervention. Values are expressed as adjusted and unadjusted odds ratios (OR) and corresponding 95% confidence intervals (CI) are reported. Generalized estimating equations method was used to take into account the clustered nature of the data (patients within physicians). All risk factors in figure 3 were included in the adjusted analysis along with the two way interaction terms with phase. The fit of the models was < 0.001.

At baseline, 68.3% (4426/6482) of high risk patients were administered a bone mineral density test as compared with 59.5% (1123/1889) of low risk patients. During the first follow-up, 78.2% (4343/5557) of high risk patients were administered a bone density test as compare with 61.4% (1088/1771) of low risk patients (Table 3). Appropriate bone mineral density testing significantly improved at the end of the 1^st ^year of the study as indicated by the unadjusted and adjusted OR and 95% CI (Figure 3). However, there are many reasons why physicians administered or did not administer bone density tests in high and low risk patients (Tables 4).

**Table 3:** Bone mineral density testing in high and low risk patients

Patient characteristics: n with characteristic/total n (%)	Baseline	Follow-up
Reason for being in high risk group		
Facture*	706/883 (80.0)	735/922 (79.6)
Age (yr)*	3564/5333 (66.8)	3384/4433 (76.3)
Family History*	362/431 (84.0)	574/648 (88.6)
Other Major*	2389/3484 (68.6)	953/1159 (82.2)
Overall High Risk Group	4426/6482 (68.3)	4343/5557 (78.2)
		
Low risk group:		
One minor or no risk factors	1123/1889 (59.5)	1088/1771 (61.4)

* Patients may have had other risk factors.

**Table 4:** Reasons why primary care physicians conducted a bone mineral density test in high and low risk patients

	Group: High Risk*	Group: Low Risk*
***Conducted a Bone Density Test***		
Total # in group	n = 4190	n = 1054
Patients request: # (%)	173 (4.1)	218 (20.7)
High risk due to fracture: # (%)	468 (11.2)	3 (0.3)
High risk (other): # (%)	1358 (32.4)	38 (3.6)
To asses risk for fracture: # (%)	2033 (48.5)	699 (66.3)
Other	158 (3.8)	96 (9.1)
***Did not Conducted a Bone Density Test***		
Total # in group	n = 1139	n = 646
Test not available: # (%)	17 (1.5)	1 (0.2)
Patient at low risk: # (%)	231 (20.3)	499 (77.2)
Patient refused: # (%)	188 (16.5)	15 (2.3)
Other: # (%)	703 (61.7)	131 (20.3)

* High risk: one major or two minor risk factors for fracture; Low- risk: one minor or no risk factors for fracture. These data were collected during Phase II.

## Discussion

Given the magnitude of the community health problem associated with osteoporosis, managing the disease must shift from specialists to family physicians. Family physicians are a trusted source of health information to their patients and have a unique opportunity to proactively prevent and treat osteoporosis in their practice for the reason that in most patient/primary care physicians relationships there are many opportunities for the physician to assess osteoporosis.

Despite the consequences of osteoporosis, the management of the disease is less than optimal [10-12,24]. For instance, Juby and De Geus-Wenceslau evaluated the presence of osteoporosis from a retrospective chart review of 311 consecutive patients over 65 years of age who were admitted to a tertiary care hospital with a diagnosis of hip fracture. In addition, chart review was conducted on 226 patients after discharge from post-surgery rehabilitation [12]. The results showed that osteoporosis was diagnosed in the tertiary care hospital on admission in 11.9% and on discharge in 15.4%. In the rehabilitation hospital, it was diagnosed in 9.7% on admission and 11.2% on discharge [12].

Given this care gap, our study was designed to improve patient care. The Quality Circle technique has been previously examined in osteoporotic patients with positive treatment outcomes. However, these circles consisted of interdisciplinary groups of physicians [25,26]. Our study demonstrated that Quality Circle methodology is an effective approach that improves family physicians' diagnosis of osteoporosis. The results showed that family physicians were more aware of their patients' risk factor status. This indicates that physicians asked more questions regarding a patient's risk factor status for osteoporosis during clinical visits. Moreover, a higher number of high risk patients received bone density testing as compared with low risk patients. However, bone density testing in low risk patients did not change dramatically following the educational intervention and the amount of testing was higher than expected. The primary reason for this finding was that family physicians wanted to formally assess the fracture risk of these patients. Fortunately, it appeared that these formal risk assessments were done prudently, given the fact that approximately two thirds of low risk patients were never administered a bone density test or were given their most recent scan three or more years following their last test. Results also suggested that following the Quality Circle meetings, there was a decrease in the difference in bone density testing in patients with a prior fracture as compared with those without a fracture. One reason for this outcome may be that physicians believed that bone density testing was not necessary in patients with fracture and that these patients should be administered therapy regardless of their bone density t-score [27,28].

Although the perceived prevalence of fracture was high, population based studies have suggest a higher prevalence then reported in the current study particularly at the spine [29-31]. This would suggest vertebral fractures are underreported and that strategies should be developed to improve reporting.

The strengths of the study are numerous and include the large number of family physicians that participated from across Canada; thus improving the generalizability of the results. In addition, the findings of our study were derived from self-audits and did not rely on physicians self-reports, which may reflect attitudes about their practice rather than true practice. Furthermore, the audits consisted of a random selection of patients from the physicians' practices. Because single-component interventions have not been shown to change clinical practice [17-19], Quality Circle methodology combined various techniques into one multifaceted intervention, which is likely more effective at changing physician awareness and behaviour [20,21]. This technique involved practice audits, feedback on performance by peers, an interactive discussion of evidence, small group physician education workshops that were led by local primary care physicians and supported by osteoporosis specialists, diagnosis and treatment reminders, and making personal learning plans for improving clinical management of osteoporosis in accordance with the OC 2002 guidelines. Finally the Quality Circle technique, consisted of short duration meetings with little financial incentives, thus the feasibility of using this approach in osteoporosis aware physicians in other settings is high.

Nonetheless, our study is not without limitations. Patients evaluated in the study were postmenopausal women and as a consequence, our results may not be applicable to men, or premenopausal women. Given that recruitment was based on a physician's interest in osteoporosis and women's health, these clinicians may have greater experience and comfort in managing the disease from the onset. In addition, this recruitment strategy may result in some selection bias, which may have influenced our findings.

Furthermore, the physicians that participated in the study were from urban settings and it has been shown that urban physicians may order more bone density tests as compared with rural physicians [32]. Moreover, a randomized control trial of physicians will be needed to confirm the current study's results. Finally, it is important to consider that clinical practice guidelines are intended to provide physicians with the current best evidence from clinical research to help them make health care decisions regarding osteoporosis; however, clinical judgment and the patient's preference, will determine if, when and what treatment is initiated. As a result, 100% adherence to the guidelines is not warranted.

## Conclusion

In conclusion, because osteoporosis is a multifactorial condition, its prevention and management are complex. It is important that physicians recognize the risks for osteoporosis and fracture and that these factors should be used to identify individuals who may benefit from bone density testing. The Quality Circle technique is an effective stepwise knowledge translation approach that increases primary care physicians' awareness and assessment of osteoporosis in accordance with the Osteoporosis Canada 2002 guidelines.

## Competing interests

**GI, LT, AG, LS**, and **FJ **did not have any competing interests. **AH **had honoraria or consultancies with Eli Lilly and Company, Merck Frosst, NPS-Allelix, Zelos Therapeutics, Servier, Pfizer Pharmaceuticlas USA, Novartis Pharmaceuticals Corporation, the Alliance for Better Bone Health (Procter & Gamble Pharmaceuticals and sanofi-aventis), and GlaxoSmithKline Consumer Healthcare. **BK **had honoraria or consultancies with the Alliance for Better Bone Health (Procter & Gamble Pharmaceuticals and sanofi-aventis). Both **DJ **and **NP **were Employees of Procter & Gamble Pharmaceuticals. **JDA **had honoraria, grants received, or consultancies with Eli Lilly and Company, Merck Frosst, Amgen Inc, the Alliance for Better Bone Health (Procter & Gamble Pharmaceuticals and sanofi-aventis), Novartis Pharmaceuticals Corporation, GlaxoSmithKline Consumer Healthcare, Servier, Roche, Servier, and Wyeth. **AP **had honoraria, grants received, or consultancies with Eli Lilly and Company, Merck Frosst, Amgen Inc, the Alliance for Better Bone Health (Procter & Gamble Pharmaceuticals and sanofi-aventis), and Novartis Pharmaceuticals Corporation.

## Authors' contributions

All authors meeting the criteria for authorship. **GI **made substantial contributions to the acquisition of data, and the analysis and interpretation of data. In addition **GI **drafted the manuscript and gave final approval for publication. **LT **made substantial contributions to the interpretation of data, critically revised the manuscript for important intellectual content and gave final approval for publication. **AG **made substantial contributions to the interpretation of data, critically revised the manuscript for important intellectual content and gave final approval for publication. **AH **made substantial contributions to the conception and design of the study and interpretation of data. Furthermore, **AH **critically revised the manuscript for important intellectual content and gave final approval for publication. **BK **made substantial contributions to the conception and design of the study and interpretation of data. Moreover, **BK **critically revised the manuscript for important intellectual content and gave final approval for publication. **DJ **made substantial contributions to the conception and design of the study and interpretation of data. In addition, **DJ **critically revised the manuscript for important intellectual content and gave final approval for publication. **NP **made substantial contributions to the conception and design of the study, interpretation of data, critically revised the manuscript for important intellectual content and gave final approval for publication. **LS **made substantial contributions to the conception and design of the study, interpretation of data, critically revised the manuscript for important intellectual content and gave final approval for publication. **FJ **made substantial contributions to the interpretation of data, critically revised the manuscript for important intellectual content and gave final approval for publication. **JDA **made substantial contributions to the conception and design of the study and interpretation of data. Moreover, **JDA **critically revised the manuscript for important intellectual content and gave final approval for publication. **AP **made substantial contributions to the conception and design of the study and interpretation of data. Moreover, **AP **critically revised the manuscript for important intellectual content and gave final approval for publication.

## Pre-publication history

The pre-publication history for this paper can be accessed here:


